# Decidual macrophages in recurrent spontaneous abortion

**DOI:** 10.3389/fimmu.2022.994888

**Published:** 2022-12-08

**Authors:** Qiu-Yan Zhao, Qing-Hui Li, Yao-Yao Fu, Chun-E Ren, Ai-Fang Jiang, Yu-Han Meng

**Affiliations:** Center of Reproductive Medicine, Affiliated Hospital of Weifang Medical University, Weifang, Shandong, China

**Keywords:** decidual macrophages, recurrent spontaneous abortion (RSA), M1/M2 balance, CD11clow/CD11chigh, the maternal-fetal interface

## Abstract

Recurrent spontaneous abortion (RSA) is defined as two or more pregnancy loss, affecting the happiness index of fertility couples. The mechanisms involved in the occurrence of RSA are not clear to date. The primary problem for the maternal immune system is how to establish and maintain the immune tolerance to the semi-allogeneic fetuses. During the pregnancy, decidual macrophages mainly play an important role in the immunologic dialogue. The purpose of this study is to explore decidual macrophages, and to understand whether there is a connection between these cells and RSA by analyzing their phenotypes and functions. Pubmed, Web of Science and Embase were searched. The eligibility criterion for this review was evaluating the literature about the pregnancy and macrophages. Any disagreement between the authors was resolved upon discussion and if required by the judgment of the corresponding author. We summarized the latest views on the phenotype, function and dysfunction of decidual macrophages to illuminate its relationship with RSA.

## Introduction

Recurrent spontaneous abortion (RSA) is an early pregnancy complication, which is defined as two or more spontaneous pregnancy loss with the same couple (1). The European Society of Human Reproduction and Embryology (ESHRE) considers it often occur continuously prior to 24 gestational weeks (2). In recent years, RSA becomes one of the formidable challenges for the doctors and infertile patients, as it may govern the fate of the whole family. However, miscarriage is a relatively common problem, occurring in 12 to 15 percent of clinically recognized pregnancies. Its risk increases with maternal age (3) and each previous prenancy losses stepwise. Patients suffered from recurrent abortion account for 1-3% (4). Large amount of data shows that RSA patients are a high-risk population for obstetrical and perinatal complications (5, 6). As the number of miscarriages has increased, the more damage to the maternal endometrium and the emergence of pelvic inflammatory disease, lead to secondary infertility. Equally, the risk of fetal growth restriction, placental abruption, premature delivery and stillbirth in future pregnancies are also raised. What can not be ignored are the following issues, such as venous thromboembolism, mental health and economic costs. Thus, closer surveillance of the RSA patients in late pregnancy must be introduced in clinical practice.

The causes of RSA are connected with anatomic defects, chromosomal abnormalities, immune dysregulation, thrombophilia, endocrine disease, infection, environmental and psychological factors (7, 8). Until now, approximately 50% of RSA cases remain elusive, leaving us away from an accurate examination and treatment. Actually, the follow-up studies of the exact etiology and pathogenesis are frequently difficult. Because of practical feasibility and ethical limitations, mouse models with higher conception rate and shorter gestation are always used in the studies of RSA (9). However, the relationship between unexplained RSA and immune system has increasingly drawn more attention in clinical practice. The latest research about a single-cell RNA sequencing showed macrophages have been observed in human yolk sac both morphologically and transcriptionally, which is essential for fetal development in early pregnancy (10). And numerous immunomodulatory therapies for RSA have been suggested.

Given the relationship with RSA, researches mostly support that the immunological factor is a prerequisite for a successful pregnancy (11). Compared to the more explicit role of NK cells in pregnancy (12), the roles of decidual macrophages in pregnancy have not been fully investigated. Macrophages are the second largest group of immune cells and account for 20 percent of the total leukocytes at the maternal-fetal interface (13). They participate in all physiological events in the female reproductive system, such as menstruation, implantation and deliver (14). On account of the polarization and plasticity of macrophages, they differentiate into specific phenotypes as a response to the microenvironmental stimuli (15, 16). The number and function of macrophages in the non-pregnant uterus are regulated by the estrogen and progesterone during the menstrual cycle (17). When the endometrium falls off at menstruation, macrophages with numbers peaking promote “wound healing” through phagocytosis and tissue remodel (18). Before implanting, macrophages are recruited to exhibit M1 phenotype to reply the inflammatory response resulting from seminal fluid. As extravillous trophoblasts (EVTs) begin to invade the decidua, decidual macrophages convert to a mixed profile of M1/M2 macrophages (19). Then, for the establishment of fetal immune tolerance, macrophages transform into an overwhelming M2 phenotype (20). By releasing proangiogenic growth factors such as interleukin 8 (IL-8), vascular endothelial growth factor (VEGF)-A and VEGF-C, M2 macrophages act as ‘bridge cells’. They jointly facilitate unique vascularization and immunosuppression in the placental microenvironment (21, 22). In the process of tissue remodeling, decidual macrophages protect embryos from phagocytosis and infection (23, 24). Therefore, decidual macrophages are indispensable in pregnancy and its dysfunction will lead to pregnancy loss.

## Phenotypes of decidual macrophages

Plasticity and polarization are landmarks of macrophages (16, 25). At the maternal-fetal interface, notable changes have occurred in the decidual macrophages. Next, we will brief the classification and possible mechanisms of macrophage in terms of its phenotype.

### M1/M2

Based on their cytokines secretion, chemokines expression and functional characteristics, decidual macrophages can be classified into two subsets: classically activated macrophages (M1 macrophages), and alternatively activated macrophages (M2 macrophages) (26–28).

Bacterial lipopolysaccharide (LPS) recognition or induction of Th1 cytokines, such as tumor necrosis factor α (TNF-α), interferon-γ (INF-γ), can drive M1 polarization of macrophages. These macrophages secrete pro-inflammatory cytokines and chemokines IL-1α, IL-1β, IL-6, IL-12, TNF-α, CXCL9, CXCL10 and express surface markers CD80, CD86, TLR-2, TLR-4, and major histocompatibility complex (MHC) class II. With the capacity of presenting antigen, M1 macrophages produce T helper type 1 (Th1) responses. It is characterized by maximizing the ability of immune cells to make cytotoxic or inflammatory reaction to viral infections, tumors or grafts. In early pregnancy, M1 macrophages promote embryo implantation and protect the fetus from infection (29–33).

Moreover, IL-4 and IL-13 directly induce M2 macrophage activation, IL-10 and transforming growth factor-β (TGF-β) make macrophages polarized toward the M2 phenotype. As the part of a polarized Th2 response, M2 macrophages are involved in apoptotic cells clearance and tissue remodeling. The release of a distinct set of chemokines, such as CCL17, CCL22 and CCL24 and their corresponding chemokine receptors CCR4 and CCR3, can also cause the recruitment of Th2 cells and amplification of polarized Th2 responses. They have immunosuppressive properties with higher levels of CD206, CD209 and CD163 expression. M2 macrophages provide an immune-tolerant environment for the fetus throughout pregnancy (29, 34–36).

Macrophages are typical plastic cells which can switch phenotypes and be subject to environmental disturbances (37, 38). Therefore, it is necessary to ensure the balance of M1 and M2 macrophages, so that the embryo can implant and develop smoothly at the maternal-fetal interface (39–42).

### CD11c^high^/CD11c^low^

Distinct from the traditional M1 and M2 macrophages, Houser classified decidual macrophages into CD11c high and CD11c low subpopulations on the basis of CD11c expression (43, 44). Moreover, Jiang et al. subdivided macrophages into three decidual subsets, CCR2^-^CD11c^LO^, CCR2^-^CD11c^HI^, and CCR2^+^CD11c^HI^ by flow cytometry analysis (45, 46). CD11c low decidual macrophages and CCR2^-^CD11c^LO^ subset expressed highly phagocytic receptors, such as CD209 and CD206 (43, 47). CCR2^-^CD11c^LO^ macrophages also specifically exhibits heme oxygenase-1 (HMOX1) (48, 49), which may be in favor of protecting the fetus from being affected by possible infections during the early pregnancy (42). While CCR2^+^CD11c^HI^ and CCR2^-^CD11c^HI^ subsets posses pro-inflammatory and anti-inflammatory characteristics respectively (46, 50). They maintain an immnue balance to facilitate the clearance of pathogen infection and keep the homeostasis of the maternal-fetal interface (**Table 1**).

**Table 1:** Different phenotypes of macrophages during pregnancy.

Macrophage type	Characteristics	Function	References
M1 macrophages	CD80,CD86,TNF-α,INF-γ, IL-1α,IL-1β,IL-6,IL-12,NO, CXCL9,CXCL10,CCR7	Pro-inflammatory; Th1 responses; Present antigen; Promote embryo implantation; Protect the fetus from infection	(20, 29–33, 39, 40, 51, 52)
M2 macrophages	CD206,CD209,CD163,IL-10 TGF-β,VEGF,CCL17, CCL22,CCL24,CCR3,CCR4	Anti-inflammatory;Th2 responses; Immunosuppressive properties; Tissue remodeling	(20, 29, 34–36, 39, 51–54)
CD11c^low^(67%)	CD206^hi^,CD209^hi^, expressed genes involved in regulating growth and development, as well as Extracellular matrix formation	Homeostatic function during placental growth	(43, 44)
CD11c^high^(33%)	CD206^low^,CD209^low^, Upregulates CD1a,CD1c,CD1d; Expressed genes associated with lipid metabolism and inflammation	Antigen processing and presentation	(43, 44)
CCR2^-^CD11c^LO^ (CD11c^low^, ~80%)	Exhibit the fewest inflammatory properties; highly express CD209 and MHCII.	Antigen presentation	(45, 46, 55)
CCR2^+^CD11c^HI^ (CD11c^high^, 10-15%)	Facilitate the clearance of pathogen infection; maintain the homeostasis of the maternal-fatal interface.	Pro-inflammatory	(45, 46, 55)
CCR2^-^CD11c^HI^ (CD11c^high^, ~5%)		Anti- inflammatory	(45, 46, 55)

## Functions of decidual macrophages in normal pregnancy

Decidual macrophages have drawn remarkable attention for their functional characteristics of plasticity and polarization (16). Throughout the maternal adaptations to pregnancy, decidual macrophages also play critical roles (56). Decidual macrophages coordinate tissue remodeling and angiogenesis, induce apoptosis of damaged cells, facilitate trophoblasts invasion and suppress inflammation (57–62). Consequently, they are indispensable in contributing to the maternal-fetal immune tolerance.

As we all know, endometrial macrophages act as determinants of uterine receptivity. Owing to the characteristics of immune tolerance, they became the research focus in the medical community (63). Gorczynski pointed out that CD200 and MD-1 have immune regulatory activity toward macrophages, which is beneficial to successful pregnancy (64). If the expression of CD200R in macrophages increases, it can stimulate the activity of indoleamine2,3-dioxygenase (IDO) (65). Thus, establishing an immunosuppressive environment is necessary for successful implantation (66). Furthermore, there are many kinds of Toll receptors on decidual macrophages, such as TLR2, TLR3 and TLR4. In response to TLRs activation, decidual macrophages facilitated the secretion of pro-inflammatory cytokines IL-1β, TNF-α, IL-6, IL-8 and the production of the anti-inflammatory cytokines IL-1RA and IL-10. Among these cytokines, IL-10 was the most easily induced. Along with the higher secretion of IL-10 increased by TLRs activation, it might help sustain immune tolerance by curbing the action of pro-inflammatory cytokines (67). In addition, the inhibitory receptors expressed on invading extravillous trophoblasts, such as immunoglobulin-like transcript 2 (ILT2) and ILT4 for human leukocyte antigen (HLA)-G (68, 69), can be combined with the decidual macrophages. As a negative signal that be delivered to the decidual macrophages, they are in favor of the production of anti-inflammatory cytokines and tolerance to the trophoblast (70).

Human decidual MMP-9^+^ macrophages can degrade the extracellular matrix (ECM) and promote endovascular trophoblast invasion, and they are enriched in the vicinity of the trophoblast invasion during early pregnancy (71). IL-33, as a cytokine of the IL-1 family, is found to be associated with Th2 and M2 polarization (72, 73). It can accelerate the development of primary trophoblasts, villous cytotrophoblast (74). Granulocyte macrophage colony-stimulating factor (GM-CSF) and macrophages colony-stimulating factor (M-CSF) are secreted from first trimester decidual cells. These activated cells can promote macrophages activation, and induce extravillous trophoblasts (EVTs) apoptosis through the caspase 3/7 dependent pathway (75). Conversely, trophoblasts-derived IL-6 (76), CXCL16 (77), and hyaluronan (HA) (78) could induce M2 macrophages polarization. In addition, macrophages could also secrete exosomes or extracellular vesicles and deliver miRNAs to affect the invasion and migration capabilities of trophoblasts, thereby participating in the occurrence of RSA (51, 79).

Placental macrophages are a special M2-like polarized phenotype, which don’t possess all properties of M2 cells. However, the expression intensity of CD163, CD80, CD11c, CCR5, CXCR4 on M2-like macrophages are lower than M2 macrophages. They were shown to regulate gap junction communication and promote decidualization (80).

Decidual macrophages may be the major APCs in the decidua (81). It is thought to be the sentinels of the immune system that initiate and regulate the immune response (82). M2 macrophages can wipe out infection by switching gene expression toward anti-inflammatory cytokines including IL-10, TGF-β and IL-1Ra (83). It also express high levels of scavenger receptors CD163, Stabilin-1 and c-type lectins receptors CD206 and CD209 (84). Phagocytosis of damaged and apoptotic cells are fundamental M2 macrophage functions, which also apply to decidual macrophages at the maternal-fetal interface.

## The relationship between decidual macrophages and RSA

**Figure 1:**
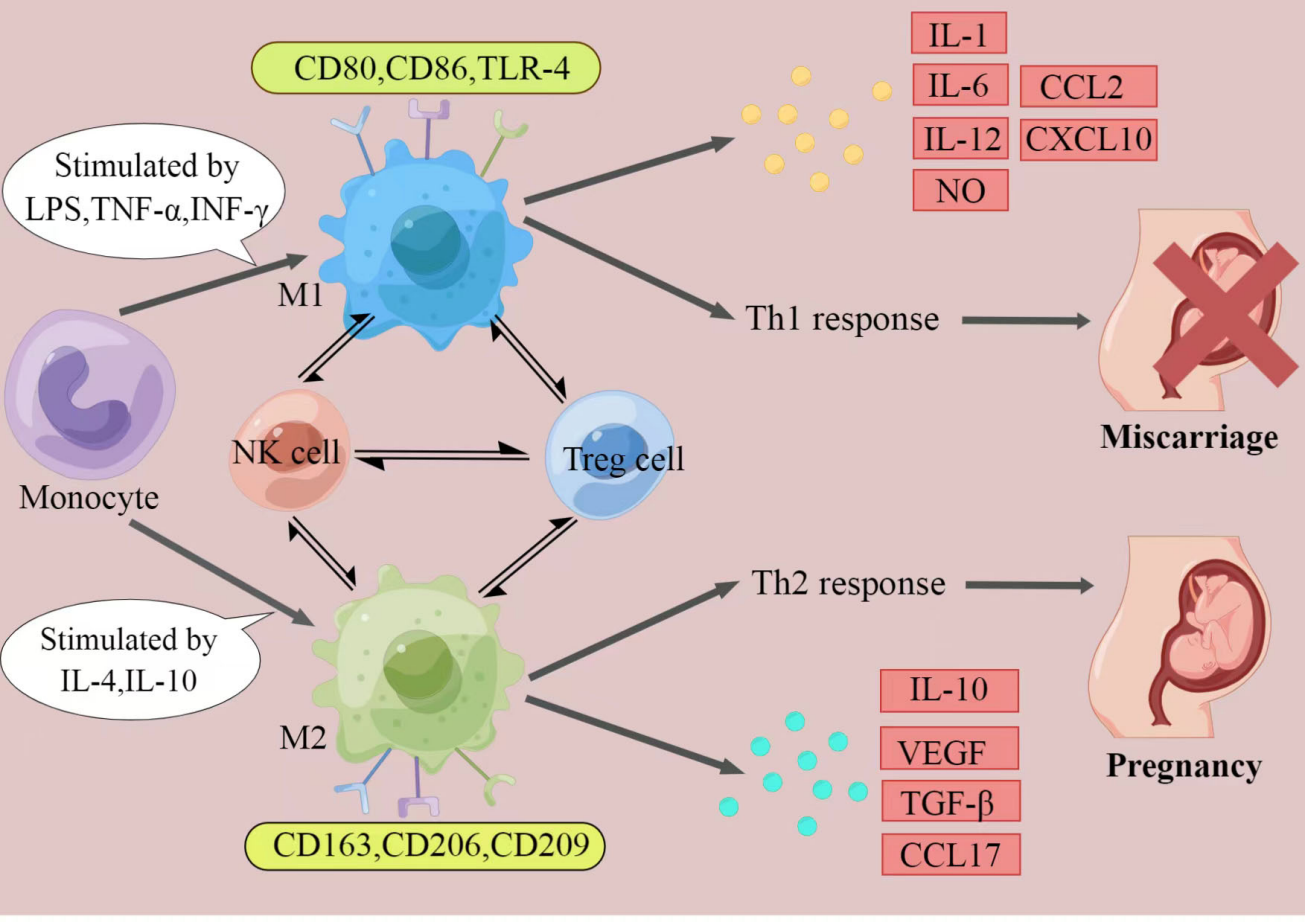
The polarization of macrophages and their characteristics. The figure displays a general principle of polarized M1 and M2 macrophage. M1 and M2 phenotypes represent two extremes of macrophage polarization and display distinct functions, thereby result in different pregnancy outcomes. In response to different stimuli, decidual macrophages undergo M1-like, or M2-like activation. M1 macrophages are stimulated by LPS, TGF-α, or IFN-γ. They express CD80, CD86, and TLR-4, secrete IL-1, IL-6, IL-12, NO, CCL2 and CXCL10, and produce Th1 responses, exert pro-inflammatory effects. In contrast, M2 macrophages are activated by IL-4 or IL-10. They express CD163, CD206, and CD209, secrete IL-10, VEGF, TGF-β and CCL17, and promote Th2 responses, provide an immune-tolerant environment for the fetus. Thus, if M2 macrophages play the major role at the maternal-fetal interface, pregnancy would continue. When M1 macrophages are absolutely dominant, it will ultimately lead to miscarriage. (Created by Figdraw).

It has been fully elaborated that RSA have an immune background. What’s the relationship between decidual macrophages and the aetiology of RSA? How do dysfunction of decidual macrophages lead to RSA (**Figure 1**)?

### M1/M2 macrophages balance

As it was mentioned above, the disturbed M1/M2 macrophages balance came to light at the maternal-fetal interface of RSA (40). It seems that the M1 subtype predominates over the M2 subtype in those cases, accompanied by pregnancy complications (85, 86). M1 macrophages can suppress epithelial-mesenchymal transition (EMT), migration, and invasion of trophoblasts by transporting miR-146b-5p to directly inhibit TRAF6 expression, thereby participating in the pathogenesis of RSA (87). ChunYan Wei have tested that JAK2 inhibitor adjusted the proportion of M1/M2 macrophages, further affecting the pregnancy outcome through the CCL2/CCR2/JAK2 pathway (88). Decreased programmed death-1 (PD-1) protein expression on decidual macrophages, accompanied with reduced programmed cell death ligand-1 (PD-L1) expression on placental villi, was observed in RSA. Meanwhile, the disturbed PD-1/PD-L1 axis induced M1 differentiation (89). Knockdown of CYP26A1 in mice uterine can decrease the number of embryo implantation. It can be also discovered that the protein levels of M1 markers TNF-α, IL-6 and CD86 were significantly decreased, thus leading to the insufficient M1 polarization (90). Additionally, in the immune atlas of RSA without chromosomal aberrations, pro-inflammatory subsets of CD11c^high^ macrophages increased remarkably (91). The present research illustrated that the abnormally increased MNSFβ expression can promote the secretion of TNF-α, inducing the polarization of decidual macrophages toward a pro-inflammatory phenotype (92). More intuitive studies on mouse experiments confirmed that Cathepsin E-deficient mice displayed compromised immune reactions with higher susceptibility to bacterial infection (93).

### Cytokines

Several studies have demonstrated that the expression of CD80, CD86 and HLA-DR, but not CD163 on decidual macrophages were higher in RSA patients compared to normal pregnancies, accompanied with higher production of TNF-α and lower secretion of IL-10 and IL-33 (40, 73, 94). In LPS-induced mice abortion model, the expression IL-1, IL-6, TNF-α, IFN-γ, IL-17a was significantly raised (95). Macrophage depletion was also proved to prevent CpG-induced embryonic resorption in an abortion mice model and in IL-10^-/-^ mice (96, 97). The experiment as early as the 18th century has confirmed that the depletion of macrophags results in the loss of pregnancy and recurrent abortion (57). CSF-1-deficient mice displayed few decidual macrophages, with lower implantation at day 7 and 8, and always had aberrant fertility with smaller size (98). On day 0.5 or day 3.5 post-coitum, injection of diphtheria toxin (DT) to Cd11b-Dtr mouse model caused implantation failure and infertility. But implantation failure can be alleviated by administration of bone marrow-derived CD11b^+^F4/80^+^ monocytes/macrophages (99). And injection on day 14 and 16 led to fetal mortality without cervix ripening (100). However, it could be alleviated by administration of RANK^+^ macrophages (101). RANKL derived from trophoblasts could make macrophage polarization to M2 by activating AKT/STAT6-Jmjd3/IRF4 signaling pathway. The knockout model of RANK^−^/^−^ mice can lead to the decreased expression of TGF-β and the increased pregnancy loss (102). Mice with uterine deficiency of high-mobility group box-1 (HMGB1) protein, showed impaired implantation and severe subfertility (103). But highly expressed HMGB1 was actively secreted by macrophages and then activated pyroptosis, leading to the occurrence and development of RSA (104).Therefore, restricting macrophages accumulation is also needed.

T-cell immunoglobulin and mucin domain containing protein 3 (Tim-3) blockade down-regulated the phagocytosis of decidual macrophages, leading to accumulation of inflammatory granulocytes and macrophages at the maternal-fetal interface (105). Therefore, high level of pro-inflammatory cytokines establishes a pro-inflammatory microenvironment and impairs normal pregnancy. It also has been indicated that dysregulation of decidual macrophages activation by regulatory T cells (Treg cells) may lead to RSA. When Treg cells regulate aberrant cell-cell contact, there will be a problem with decidual macrophages. The abnormity of decidual macrophages was indicated to be regulated by Treg cells through aberrant cell-cell contact and TGF-β secretion (106). Moreover, Jiayu Wang’s studies showed that abnormally expressed USP2a may be found in the placental villous samples of RSA patients. Further studies have confirmed that TGF-β could collaborate with USP2a to promote trophoblasts migration and invasion *via* its interaction with TGFBR1 (107). Thrombospondin1 (TSP1) needs to interact with CD36, CD47 and heparin sulphate proteoglycanto enhance the ability of macrophages (108). They are engaged in regulating IL-10 secretion and boost the tolerance of the immune system at the maternal-fetal interface (109). Thus, low expression of TSP1 along with decreased IL-10 could appear in RSA.

## Therapies

Owing to the uncertainty of the pathogenesis, the likelihood of recurrence, recent studies suggest that various treatment of RSA may work (110, 111). Clinicians often use progesterone to support or supplement the pregnancy, by oral, vaginal, intramuscular, or other ways. It is considered to be essential for successful embryo implantation. But now, it is increasingly becoming the psychological support to patients (112). Besides, all treatments for RSA are almost based on immunomodulation for their effects (2, 113). A meta-analysis of the treament of APS-related RSA showed that aspirin plus low-molecular-weight heparin (LMWH) can significantly reduce the rate of repeated pregnancy loss (114). Another study proved the combination of anticoagulant and anti-inflammatory could contribute to a better pregnancy outcome (115). Prednisone, hydroxychloroquine (116) and cyclosporine A (117) are also part of the clinical therapy regimen of RSA.

When a semi-allogeneic fetus appears at maternal-fetal interface, maternal tolerance is required to avoid the miscarriage. Patients with RSA may lack this capacity. Therefore, alloimmunization was born in response to the condition. It has been suggested that the effect of lymphocyte immunotherapy (LIT) was probably positive. And a higher success rate was likely observed in those immunized with paternal lymphocytes (118). Some experts recommend immunotherapy before and during pregnancy with low dose of lymphocytes. It can break the balance between Th1 and Th2 cytokines, reducing the level of Th1 cytokines (IL-2, INF-γ, TNF-α, and IL-6), while increasing the level of Th2 cytokines (IL-4, IL-10) (119). As opposed to “active immunization” with allogenic lymphocytes which was introduced previously, intravenous immunoglobulin (IVIg) was termed as “passive immunization”. The effect of IVIg on Treg/Th17 cells ratio enhances Treg cells function, and thereby improve the live birth rate in pregnancy to some extent (120). The RSA mice models with intraperitoneally administration of G-CSF certified that the absence of G-CSF weakened the inhibitory effects on macrophages, leading to more M1-type differentiation and overexpressing NLRP3 inflammasomes at the maternal-fetal interface. It implies that G-CSF may improve pregnancy success rate by modulating the inflammatory state (121).

It is worth noting that immunotherapy is not a panacea for treating all patients with RSA. The choice of therapeutic plans should have certain indications (122). For unexplained RSA, we should make efforts to seek the pathogenesis. Testing for inherited thrombophilia and hyperhomocysteinemia should be performed. If necessary, screening for immunological factors such as Human Leukocyte Antigen (HLA), cytokines, antinuclear antibodies (ANA), Natural Killer (NK) cells, anti-HLA antibodies and antisperm antibodies, Lupus anticoagulant (LAC), Anticardiolipin antibodies (ACL) and anti-β2 glycoprotein-I antibodies (β2-GPI). So, further research in personalised treatment options is warranted.

## Conclusion

Take together, polarized macrophages can influence the reception of maternal to embryo through the secretion of various cytokines and chemokines. The specific etiology and pathogenesis among them is very complicated, which is an emerging field that needs to be explored urgently. How to maintain M1/M2 macrophages balance? Even we know the correlation of decidual macrophages with RSA, what can we do to help them? Collectively, there are a large number of challenges to be overcome, and further efforts are needed.

## Data Availability

The original contributions presented in the study are included in the article/supplementary material, further inquiries can be directed to the corresponding author/s.
